# *Mycobacterium bovis* Infection in Free-Ranging African Elephants 

**DOI:** 10.3201/eid2703.204729

**Published:** 2021-03

**Authors:** Michele A. Miller, Tanya J. Kerr, Candice R. de Waal, Wynand J. Goosen, Elizabeth M. Streicher, Guy Hausler, Leana Rossouw, Tebogo Manamela, Louis van Schalkwyk, Léanie Kleynhans, Robin Warren, Paul van Helden, Peter E. Buss

**Affiliations:** Stellenbosch University, Cape Town, South Africa (M.A. Miller, T.J. Kerr, C.R. de Waal, W.J. Goosen, E.M. Streicher, G. Hausler, L. Kleynhans, P. van Helden);; South African National Parks, Skukuza, South Africa (L. Rossouw, T. Manamela, P.E. Buss);; Skukuza State Veterinarian Office, Skukuza (L. van Schalkwyk);; South African Medical Research Council Centre for Tuberculosis Research, Cape Town (R. Warren)

**Keywords:** African elephant, endemic bovine tuberculosis, GeneXpert Ultra, Kruger National Park, *Loxodonta africana*, *Mycobacterium bovis*, tuberculosis and other mycobacteria, South Africa, bacteria

## Abstract

*Mycobacterium bovis* infection in wildlife species occurs worldwide. However, few cases of *M. bovis* infection in captive elephants have been reported. We describe 2 incidental cases of bovine tuberculosis in free-ranging African elephants (*Loxodonta africana*) from a tuberculosis-endemic national park in South Africa and the epidemiologic implications of these infections.

Tuberculosis (TB), caused by the human pathogen *Mycobacterium tuberculosis*, is a recognized disease in human-managed and wild Asian elephants (*Elephas maximus*) and African elephants (*Loxodonta africana*) (*1*–*3*). Previous findings demonstrate the importance of human-elephant interfaces for transmission. However, range countries for African and Asian elephants also have high burdens of bovine TB, caused by *M. bovis*. The World Organisation for Animal Health (OIE) records cases of bovine TB; in the 49 elephant range countries in Africa and Asia, only Namibia is declared free of *M. bovis* (*4*). Therefore, the paucity of cases of *M. bovis* infection in elephants is unexpected. The lack of *M. bovis* cases in elephants may be caused by rare or sporadic exposure, innate resistance of the species, or limited surveillance, especially in environments to which bovine TB is endemic.

Kruger National Park (KNP) in South Africa has recorded *M. bovis* infection in >20 wildlife species and is considered a bovine TB−endemic area. Although cases of *M. bovis* infection have been reported in other large herbivores, such as black rhinoceros (*Diceros bicornis*) and white rhinoceros (*Ceratotherium simum*) (*5*,*6*), only 1 case of *M. tuberculosis* infection has been found in an elephant in KNP (*3*), despite hundreds of individual animals examined during 1967–1994 when elephants were harvested (*7*). After the discovery of an *M. tuberculosis*–infected adult bull elephant in 2016 (*3*), opportunistic sampling of elephants was implemented by park veterinarians. 

In May 2018, a young bull elephant (E1; estimated age 18–20 years) was fatally shot in the southern part of KNP. In addition, a young bull elephant (E2; estimated age 3 years) in KNP was euthanized in October 2019 after being found moribund. Postmortem examination of E1 revealed rare small, consolidated masses in the lung. Elephant 2 had several focal firm masses (1–2 cm^2^) scattered in the lung containing caseous material and some mineralization. We took representative samples from the peripheral (prescapular, inguinal, popliteal), head (parotid, retropharyngeal), thoracic (tracheobronchial), and abdominal (mesenteric) lymph nodes; lung lesions were also sampled. We froze samples at −20°C and transported them for mycobacterial culture and speciation in the Biosafety Level 3 laboratories at Stellenbosch University (Cape Town, South Africa) and ARC-Onderstepoort Veterinary Institute (Pretoria, South Africa).

We prepared tissues for mycobacterial culture as previously described (*8*) using the BD BACTEC MGIT 960 Mycobacterial Detection System (Becton Dickinson, https://www.bd.com). We performed mycobacterial speciation by region-of-difference PCR (*8*) and spoligotyping (*9*) on Ziehl-Neelsen stain positive cultures. We isolated *M. bovis* from both tracheobronchial lymph node cultures from E1. Culture results from E2 confirmed the presence of *M. bovis* in 2 lung samples in 2 different laboratories, and in a tracheobronchial lymph node cultured in the second laboratory. We characterized the *M. bovis* spoligotype pattern from E1 as SB0121, which is the most common strain found in KNP (*9*) (Table). In contrast, in E2 we found a novel spoligotype pattern (SB1681) not previously reported in KNP (Table). However, this pattern varied by 1 spacer, and it is possible that this strain evolved from the common SB0121 strain in KNP.

**Table:** *Mycobacterium bovis* information and spoligotype patterns for isolates from 2 African elephants, South Africa*

Sample no.	Year	Species	Spoligotype pattern	Spoligotype no.
E1	2018	*M. bovis*	■■χ■■■■■χ■■■■■■χ■■■■χ■■■■■■■■■■■■■■■■■χχχχχ	SB0121
E2	2019	*M. bovis*	■■χ■■■■■χ■■■■■■χ■■■■χ■■■■■■■■■χ■■■■■■■χχχχχ	SB1681

*Isolates were identified by mycobacterial culture and speciation.

The finding of *M. bovis* infection in 2 free-ranging African elephants in KNP has significance for other elephant populations in bovine TB–endemic areas. In South Africa, the Department of Agriculture, Land Reform, and Rural Development has the authority to place *M. bovis*–infected premises under quarantine. Previously, elephants and rhinoceros were excluded from movement restrictions placed on other species in bovine TB–endemic populations. However, our findings require revisiting this assumption and investigating disease transmission risks for these species. Kerr et al., in a retrospective study investigating antibodies to *M. tuberculosis* complex antigens in KNP elephants, has estimated seroprevalence at 6%–9% (*10*), suggesting that *M. bovis* and possibly *M. tuberculosis* infection is more common in KNP than previously thought. However, no additional samples were available to confirm infection in the individual elephants studied. It is more likely that these seropositive responses in elephants represent infection with *M. bovis* because all the cases of TB in rhinoceros to date have been due to *M. bovis* infection (*5*,*6*). Isolation and speciation of pathogenic mycobacteria are essential for understanding the epidemiology of these infections, especially in areas where human–livestock–wildlife interfaces occur.

Our findings emphasize the importance of surveillance using molecular and bacteriological tools for detection of bovine TB in elephants because serologic assays and visual assessment of gross and histopathological lesions cannot differentiate between infection with *M. bovis* and *M. tuberculosis*. In areas where elephants may have indirect (through shared forage and water sources) or direct contact with animal and human populations with high burdens of *M. bovis* and *M. tuberculosis*, such as African and Asian range countries, it is crucial that surveillance and diagnostic tools are readily available to distinguish these pathogens to improve our understanding of epidemiology of TB at human–livestock–wildlife interfaces.
